# Multifunctional Integration of Optical Fibers and Nanomaterials for Aircraft Systems

**DOI:** 10.3390/ma16041433

**Published:** 2023-02-08

**Authors:** Carlos Marques, Arnaldo Leal-Júnior, Santosh Kumar

**Affiliations:** 1 i3N & Physics Department, University of Aveiro, 3810-193 Aveiro, Portugal; 2 Mechanical Department and Graduate Program in Electrical Engineering, Federal University of Espírito Santo, Espírito Santo 29075-910, Brazil; 3 Shandong Key Laboratory of Optical Communication Science and Technology, School of Physics Science and Information Technology, Liaocheng University, Liaocheng 252059, China

**Keywords:** structural health monitoring, aviation, smart materials, optical fiber sensors, aircraft security, flight-by-light

## Abstract

Smart sensing for aeronautical applications is a multidisciplinary process that involves the development of various sensor elements and advancements in the nanomaterials field. The expansion of research has fueled the development of commercial and military aircrafts in the aeronautical field. Optical technology is one of the supporting pillars for this, as well as the fact that the unique high-tech qualities of aircrafts align with sustainability criteria. In this study, a multidisciplinary investigation of airplane monitoring systems employing optical technologies based on optical fiber and nanomaterials that are incorporated into essential systems is presented. This manuscript reports the multifunctional integration of optical fibers and nanomaterials for aircraft sector discussing topics, such as airframe monitoring, flight environment sensing (from temperature and humidity to pressure sensing), sensors for navigation (such as gyroscopes and displacement or position sensors), pilot vital health monitoring, and novel nanomaterials for aerospace applications. The primary objective of this review is to provide researchers with direction and motivation to design and fabricate the future of the aeronautical industry, based on the actual state of the art of such vital technology, thereby aiding their future research.

## 1. Introduction

More than even sustainability is a strong word used in different areas of research in order to orientate, in the right way, the progress of digitalization for obtaining unique and smart high-tech qualities in different industrial sectors. One of the most critical sectors is aerospace, where the aeronautical industry needs to align the progress, sustainability, and photonics. In this way, advances in smart sensors and nanomaterials are crucial. In the last year, photonics has brought a growing role in aeronautical and aviation fields, from heads-up displays to onboard optical fiber networks, improving aircraft monitoring and maintenance in a means that is not possible with copper-based electrical systems. Although numerous developments in commercial aircrafts take place in zones of a plane that travelers do not see, still some enhancements that occur inside the passenger cabin are required, such as LED illumination, communication between crew and passengers, or electrochromatic dimming of the windows.

Before, the implementation of the “fly-by-wire” perception has significantly reduced the weight and complexity of traditional electro-mechanical systems, also increasing the stability and safety in airplanes [1]. Typical commercial or military aircrafts involve a huge number of control and monitoring systems, demanding a big quantity of sensors distributed along the aircraft. From a copper cable network, the output of electrical signals is routed for the commanding computers, which activate the actions. Over the last few years, the aeronautical industry has changed a lot, due to the climate concerns growing, in order to decrease emissions and progress on fuel efficiency. For that, a hard job has been achieved by the progress of composite fuselages to replace the traditional metallic materials, thus building lightweight aircraft models. While this serious change also takes reduced manufacturing and operating costs, composite structures subject avionic systems to a bit of a severe level of lightning-induced voltage and current. Actually, one of the main factors leading to airplane failures is the subsequent electrical wire damage [2]. In this context, optical technologies have endorsed the migration of copper harness to optical fiber-based systems, following the “fly-by-light” perception, due to the high electromagnetic immunity [3,4], where such a concept has been brought under huge affirmation, in terms of reliability and security [4]. Further, the modern aircrafts have higher operational requests driven by the increase, not only in terms of safety on critical systems for flight and engine control, but also in non-safety critical systems, such as cabin environmental control systems, structural and engine health monitoring (EHM) systems, structural health monitoring (SHM) [5], etc., which are answered more than just in the optical domain [4,5,6,7,8].

Presently the optical fiber sensors (OFS) are seen, in general, by the scientific community and industrial and end-user communities to be the technology with the maximum potential for the continuous real-time monitoring of airplane structures in different topics [4,5,9,10,11] from flight environment sensing or sensors for navigation to pilot vital health monitoring. The additional potential for integrating OFS into composite materials during the layup process would also enable the monitoring of composite structures during their entire life cycle, improving their safety, cost efficiency, reliability, and also spreading the operational life. In addition, OFS are largely known by their particular characteristics, such as electromagnetic fields immunity, compactness, multiplexing capabilities, passive operation, biocompatibility, and chemical stability [11].

The particular profits to measure and report data by using such sensors include: reducing the aircraft weight replacing bulky systems, decreasing costs (as they can be cheaper than other types, due to particular characteristics, such as multiplexing or multipoint features) [11], and providing predictive data that can help the flights become further fuel-efficient. Such sensors and systems could offer capacities and measurements that are not conceivable with other components [5,9,10]. In additional, OFS can be installed in a smaller portion of time, compared with their alternatives. From the nature of how optical fiber works, numerous sensors can be combined using a unique optical fiber, which means non-complexity installation involving just one fiber to be attached to the aircraft structure and, consequently, linked to the data acquisition equipment.

**Figure 1:**
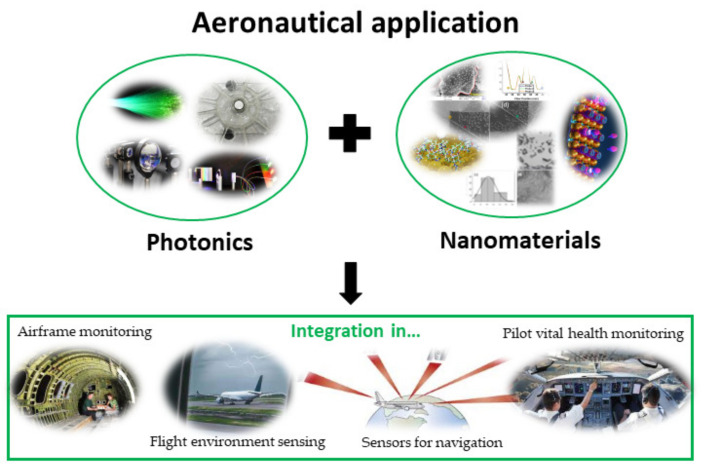
Illustration about the overall content considering the problems and the advances of photonics and nanomaterials for aeronautical sector.

In this way, this paper reviews some critical aspects, in order to see that the future is expected to see an enlarged effort on the use of OFS and nanophotonic materials potential in the aircraft to expand on the industry’s safety and also in the humans that are linked with such industry, such as flight attendants, pilots, the airport maintenance crew, etc., as well as to extend the aircrafts’ lives, decrease the requirement for time consumption and expensive maintenance actions, and increase the flights’ efficiency, in order to pave the way for sustainable structures and transportation (see Figure 1).

## 2. Airframe Monitoring

As is well-known, the strong use of composite structures in the aerospace industry over the last few decades, with newer aircraft such as Boeing 787 and Airbus A350 made largely (respectively 50% and 503 [12,13]) of composite materials, brought considerable number of new successful contributions to the progress of the SHM field for composite structures. This development can be attributed to several research performed until now by many researchers to achieved materials with particular mechanical properties, for instance, high strength-to-weight ratio and corrosion resistance. Therefore, such use of composite structures in aircraft permit us to condense the operational costs, due to less fuel consumption and less maintenance protocols. Although several advantages can be reached when is used composite structures for aircrafts, their performance or comportment pose challenges, namely in the inspection and monitoring of damage in the aircraft structure, where the damage may be present inside, but not discernable upon visual checkup of the outside layers from aircraft structure through maintenance protocols.

**Figure 2:**
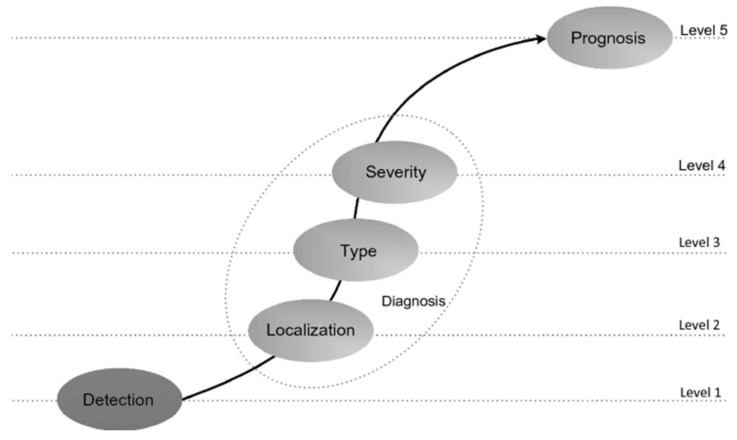
Diagram with four SHM levels for a full damage diagnostic to obtain the prognostic.

In aeronautical and aerospace industries the application of SHM has still immature [14] and specifically its application to composite structures is very challenging. For a real aircraft maintenance situation, a full damage diagnostic on all four SHM levels, rather than a single level (see Figure 2), is required, it means: (a) damage detection, (b) damage localization, (c) damage type identification, and (d) damage severity.

The SHM with success application to aircraft needs two situations: be a profitable process for the airline operators by reducing economic losses caused by unproductive downtimes and provide accurate and reliable data about the condition of the structure, growing the security of critical components to consequently avoid disasters. Therefore, from industry and academia, an extensive range of potential SHM technologies is being developed to satisfy these conditions [15], where the most capable options are acoustic emission methods, micro-electro-mechanical systems (MEMS), electrical strain gauges, crack wires, optical-based technologies, and comparative vacuum monitoring [16]. However, from the extensive works performed until now, the optical technologies have been the future for the aircrafts’ airframe, in terms of SHM.

OFS, such as the ones based on fiber Bragg grating (FBG), and optical measurement systems have previously found a range of exciting practical applications in the damage and load monitoring of aircraft composite structures, namely in ground tests and design. As well-known from the literature [9], the FBG sensor’s operation principle is based on the Bragg wavelength shift, related to variations on grating period and refractive index (RI). Generally, these variations occur due to strain (Δε) and temperature (ΔT) effects on the FBG, as shown in Equation (1): the temperature variations lead to thermal expansion (which leads to a grating period variation, proportional to the temperature and thermal expansion coefficient, α) and thermo-optic effect (RI variation proportional to the temperature and thermo-optic coefficient, ξ). The strain leads to the variation on the grating period from the applied strain and the photoelastic effect (RI variation proportional to the strain and photoelastic constant, P_e_).

(1) $$\Delta \lambda_{B}=\left[(1-P_{e})\varepsilon +(\alpha +\xi )\Delta T\right]\lambda_{B}.$$

Some aircraft are in action with integrated OFS networks achieving measurements during flights. Airbus reported in 2013 that the long-term idea is that all new aircraft will fly with distributed FBG optical sensors [17]. Indeed, some of the developed solutions are considered relatively mature, at a technology readiness level (TRL) of 5–6. For instance, it has proved the viability and effectivity using FBG-based local damage detection, when applied to composite parts of aircraft as bonded repairs [1]. Nevertheless, the broader acceptance of the use of such sensing systems is still hindered by the following issues: sensor performance when embedded, capability of detection, maintainability, available interrogation equipment as low-cost solution, lack of standardization, and certification framework.

**Figure 3:**
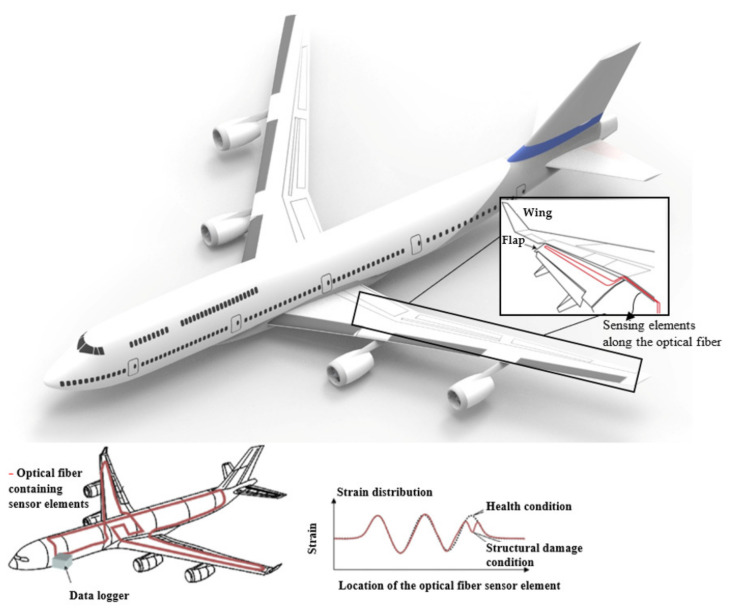
Schematic representation of sensor elements along the optical fiber that can be useful to measure the elongation of an aircraft flap, strain distribution, and structural damage condition and all data collected by data logger.

One of the main challenges for multipoint (FBG-based) and distributed detection techniques, from the point of view of the ability to detect damage, continues to be the development of controlled methods to monitor the main parameters related to the onset and growth of damage over time of large structures, with adequate physical and spatial resolution, even when the position of the damage is not known precisely enough. All aeronautic companies and entities address the issues of reducing operating costs, while achieving greater aerodynamic efficiency and improving the safety and the reliability of future aircraft. Aircraft health monitoring involves using sensors to monitor the integrity of the advanced structural materials that are expected to become the backbone of next-generation airframes. Graphite-reinforced composites give the potential for larger strength and stiffness-to-weight ratio than aluminum alloys. OFS offer the potential for high-density sensor coverage with slight weight penalty. Numerous sensors used for load, strain, and shape monitoring [9,10,11] for wing shape measurement or landing gears have been reported. Optical devices for applications relating to the health monitoring of composite materials have also been reported [18]. Graphite-epoxy panels were manufactured with integrated optical fibers of different types. The panels were thermally and mechanically tested to assess composite strength and sensor durability. The experimental results to evaluate the ability of surface-mounted and embedded optical fibers to measure strain and temperature were reported. The OFS performance was compared directly by the results from traditional instrumentation. Such experimental results indicate that OFS, integrated with composites, have potential applications for monitoring the structural integrity of critical parts in aerospace and aeronautical vehicles. In this way, fibers were successfully embedded in high-performance graphite/polyimide and graphite/epoxy parts, achieving the potential for aircraft structural applications [18] and demonstrating the use of single-mode optical fibers to measure strain and temperature in composites. Figure 3 shows a representative overview about sensor elements along the optical fiber that can be useful to measure the elongation of an aircraft flap, wings shape, strain distribution, and structural damage condition, as well as all data collected by a data logger. So, it is possible that the understanding of composite aging uses the capability to measure and correlate chemical and physical property changes. From this information, predictive decisions can be made, taking into account the state of health of a composite part and understanding what procedures to do as a requirement, thus avoiding catastrophic failure.

## 3. Flight Environment Sensing

### 3.1. Critical Environmental Sensing

Different engineering structures are exposed to environmental conditions related to the operation, or even the natural aspects, of the environment. The environmental sensing plays an important role not only on the structural analysis, but also in the navigation and control systems [19]. It is also important to mention that the dynamics of the environmental conditions (such as temperature and relative humidity) directly affect the operational and structural analysis in aircrafts in both corrective and predictive maintenance conditions [20]. Furthermore, the environmental monitoring is critical for the stress/strain sensors, since many sensors present temperature cross-sensitivity, where the structural failures can be also related to the temperature distribution dynamics [21].

In aircraft operation, the environmental monitoring is crucial for the navigation and instrumentation equipment, where the Air Data System is responsible to provide the flight data to the crew [22]. Considering an important technology for the system’s navigation, especially on the external measurements of speed, the Pitot tube technology is widely employed [23]. Such a sensor uses the air pressure in dynamic and static intake conditions, in which the operation in the harsh environments of the flight conditions can lead to freezing of the device’s orifice that can ultimately lead to critical issues in the sensor reading and even accidents [22]. To that extent, the Federal Aviation Administration provides instructions for the atmospheric conditions that can lead to such critical issues in the instrumentation, which also indicates the necessity of measuring the atmospheric/environmental conditions in-flight [22].

It is also important to notice that the environmental conditions, including temperature, moisture concentration, and pH, can lead to increases in maintenance costs and downtime, due to corrosion in different parts of the aircraft [24]. For this reason, environmental analysis also plays an important role in the structural defects, due to environmental effects, mainly the corrosion of the aircraft structure and components [25]. In summary, the corrosion is an electrochemical deterioration of metallic structures, which is caused by the chemical reactions of the material with the environmental conditions [26]. The extreme environmental conditions in aircraft operation (such as the freezing conditions in conjunction with extreme heating of some parts) exposes the aircraft structure and components to a variety of corrosion mechanisms [25]. Such a scenario includes additional criteria on the aircraft materials selections, where the design can be performed while also considering the corrosion resistance [24]. However, achieving such corrosion resistance in conjunction with the critical performance aspects, such as stiffness, weight, and strength, lead to the multi-objective problem of optimization in the material features that may not be completely fulfilled with a single material.

To that extent, the corrosion detection on the structures and components have been investigated [24]. In general, the detection is based on vision systems, which have drawbacks on the analysis of inaccessible areas. For this reason, the use of environmental sensors for indirect corrosion detection, where the environmental or local conditions can be used to correlate the corrosion parameters, such as location, time, and rates [27]. In this case, the environmental parameters monitoring, namely temperature, humidity, and even chemical compounds concentration, can be associated with corrosion, as well as its early detection or estimation [28]. Despite the influence of the atmospheric pollutants and compounds, such as acid sulfates and acid chlorides, as well as sea salts diffused into the moisture, the moisture detection and temperature play critical role in the corrosion analysis [24]. In the dynamic measurement of moisture, the time at which there are atmospheric conditions for surface layer of moisture is known as the time of wetness, and it is used as a factor for corrosion growth and initiation [28]. In addition, the temperature is an important parameter in the corrosion analysis, since it not only relates to the corrosion initiation, but also on the type of the corrosion [24]. Different temperature thresholds were analyzed in the literature for their correlation with the type of corrosion, which indicate the necessity of continuous temperature monitoring in aircraft structures and components, especially the ones with direct contact with moisture or atmospheric pollutants [24].

**Figure 4:**
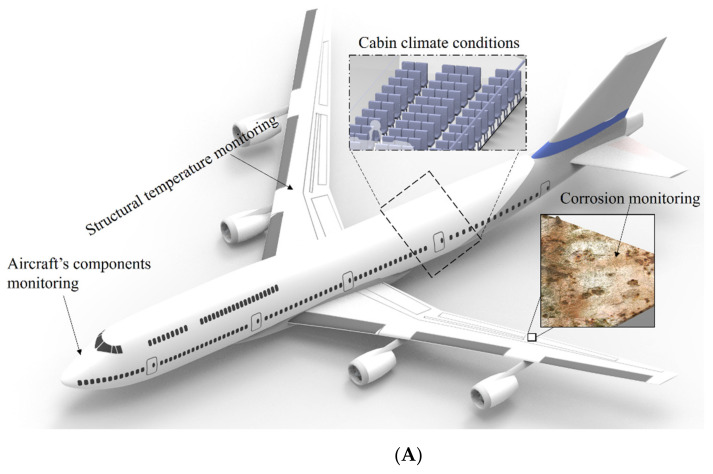

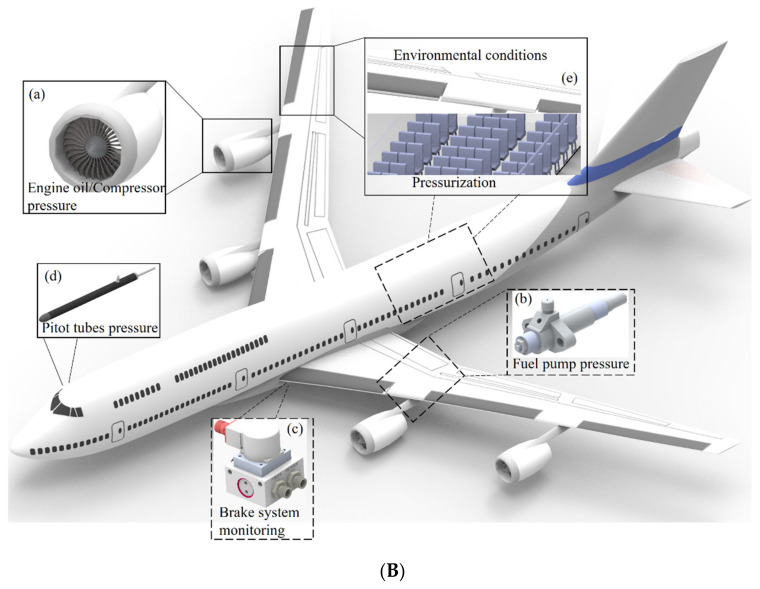
(**A**) Schematic representation of sensors positions for temperature and moisture assessment. (**B**) Schematic representation of sensors positions. (a) Engine oil pressure, compressor pressure, (b) Fuel pump pressure for fuel regulation, (c) Hydraulics braking system, (d) Air data measurement in Pitot tubes, (e) Environmental air conditioning and pressurization.

It is also important to mention that the climate conditions are important in the aircraft applications, not only on the external environmental conditions’ assessment, but also the internal environmental conditions, since the thermal comfort in cockpit or by the crew members and passengers (in the case of commercial flights) are important parameters [29]. In many cases, there is the exposure to long periods at a seated position for the crew members and passengers, which can lead to skin maceration and general injuries, due to the microclimate conditions [30]. In the literature, three regions of thermal comfort in microclimate conditions (i.e., interface between the limb and the seat) were defined, and such regions include the comfort (temperatures from 29 °C to 34 °C and relative humidity below 70%), neutral comfort (temperatures from 27 °C to 36 °C with relative humidity below 80%), and discomfort (temperatures lower than 27 °C or higher than 36 °C with humidity higher than 80%) [31]. Therefore, the applications of temperature and humidity sensors can be used as important indicators of the thermal comfort in the cabin or in the interface between the limb and the seat. Considering the necessity of temperature and humidity sensors in different positions and scenarios in an aircraft instrumentation, Figure 4A presents the schematic representation of the sensors’ positions, where their significance at each application/position was already discussed.

Among different sensors technologies, optical fiber-based sensors are a growing research field in the sensor community, due to their advantages, such as compactness, electromagnetic fields immunity, passive operation, multiplexing capabilities, chemical stability, and biocompatibility [32], as mentioned before. For these reasons, they are used for applications in different areas, such as industry [33], SHM [34], biochemical [35], and medicine [36]. A high versatility is found in OFS, since many approaches were employed throughout the years, where the intensity variation [37], fluorescence/absorbance [38], long period gratings [39], FBGs [14,40], non-uniform gratings [41], nonlinear effects [42], specklegrams [43], interferometers [44], and surface plasmon resonance [45] sensors are generally employed. As an important advantage of optical fiber sensing approaches (related to their material features, which include a flexibility, compactness, and chemical stability), such sensors are able of being embedded in rigid and flexible structures [46], as well as the integration in different dyes [47] and dopants [48]. The embedment or integration of different materials in OFS is especially important in humidity sensors development using silica optical fibers, since such an optical material is not intrinsically sensitive to humidity/moisture absorption [49].

An interferometer-based approach for humidity assessment uses a Mach–Zehnder interferometer (MZI) fabricated from a taper, where a composite film composed of graphene oxide and PVA is coated on the optical fiber sensor [50]. The applied coating is sensitive to relative humidity variation, in which there is a RI variation as a function of the environmental humidity, which leads to the possibility of humidity sensing, due to MZI transmitted spectrum variation as a function of the RI. In another interferometer sensor for humidity measurement, a Fabry–Perot interferometer (FPI) is proposed in [51]. In this case, the interferometer’s cavity is obtained on the tip of the fiber, where a Ti_3_O_5_ thin film (168 nm thickness) with an additional humidity-sensitive film with 1621 nm thickness made of SiO_2_, which is enclosed with another Ti_3_O_5_ film with 168 nm thickness. Thus, the Ti_3_O_5_ films are used as reflective surfaces to create the FPI cavity, whereas the SiO_2_ film presents variations in the RI as a function of the relative humidity, where such RI variations lead to a wavelength shift on the FPI’s reflected spectrum. Similarly, the use of extrinsic FPI for humidity measurement can also be achieved using optical adhesive or polymer films that present swelling with the moisture absorption, which also lead to spectral variations of the FPI [52].

Considering the FBG sensors developments in humidity assessment, the use of silica optical fibers is also related to the optical fiber coating with different humidity-sensitive materials [53]. To that extent, the FBG can be coated with polymer films with humidity sensitivity, where the film swelling due to the moisture absorption leads to an increase in the strain on the fiber, which leads to a Bragg wavelength shift in the FBG. In this case, the use of PEG/PVA composite was investigated in [54] for the humidity sensitivity using the aforementioned principle. It is also worth noting that the cladding removal of the FBG (using chemical process such as the etching [55]) results in a sensitivity of the FBG with the external RI, which leads to the possibility of using thin films with RI variation as a function of the relative humidity for FBG-based humidity/moisture assessment approach.

In contrast with the humidity sensing principles using silica optical fibers, the use of polymer optical fibers (POFs) provides advantageous features in some applications, due to their inherent sensitivity to humidity [56]. For this reason, intensity variation-based sensors for humidity assessment were evaluated using the polymer swelling and material features dependency as a function of the humidity as the sensing principle [57]. In addition, the advances in polymer processing and major breakthroughs in FBG inscription led to the development of FBGs in POFs, the so-called POFBGs [58]. In such developments, the use of FBGs in polymethyl methacrylate (PMMA) POFs result in a sensor intrinsically sensitive to relative humidity and moisture, where there is no need for coating the optical fiber with sensitive materials [59]. It is also important to mention that etching treatments, as well as the diameter reduction of POFs, can lead to the improvement on the response time of these sensors in which the real-time moisture and humidity assessment can be achieved [60]. Furthermore, heat treatments can be applied in the POFs to obtain an insensitivity to temperature variations, as well as hysteresis reduction, in order to extend the performance of POFBG-based humidity sensors [61]. Moreover, the flexibility in POFs fabrication resulted in the possibility of developing POFs with different transparent polymers [62,63]. For this reason, POFBGs with tailored properties were developed to achieve high humidity sensitivity using intrinsic POFBG sensors [49].

As another critical parameter for environmental sensing, temperature sensors are mandatory for such assessment. If the aircraft applications are considered, there is a high range of temperatures for the sensors, where there are temperatures above 1000 °C in regions close to the engines and thermal equipment and temperatures below 0 °C for structural analysis for in-flight conditions [64]. In both scenarios (high and low temperatures), the OFS were already employed, where the stability of material properties at low temperatures already shows the possibility of using such fibers even in cryogenic applications [65]. Considering the low temperatures obtained on in-flight conditions, conventional OFS, such as interferometers [66], distributed temperature sensing [67], and FBGs [68], can be used using their intrinsic sensitivity to temperature variations along the fiber. In addition, the embedment of the optical fibers in different materials (with high thermal expansion coefficient) can extend the temperature sensor performance, especially in terms of sensitivity and resolution [69].

If high temperature applications are concerned, applications with temperatures close to the glass transition temperature or the processing temperatures of glass material (silica optical fibers) need the evaluation of the sensors, due to the variations in silica material features at such temperatures [70]. To that extent, the application of sensors based on fluorescence was proposed using the optical fiber coating in different photoluminescent materials, such as yttrium aluminum garnet (YAG), sapphire, and MgAl_2_O_4_, due to their resistance to high temperatures [71]. The fluorescence intensity ratio is commonly used in such applications, where the photoluminescent materials present fluorescence as a function of the temperature at a specific wavelength [72]. In this approach, a ratio between the intensity in the wavelength at which the fluorescence occurs and the intensity of a reference wavelength (without the fluorescence) is obtained and analyzed as a function of the temperature [73].

Another important breakthrough in high temperature development is micromachining, especially using the femtosecond (fs) laser for the fabrication of micro-structured devices in optical fibers, such as interferometers [74]. These devices can be used in high temperatures, but below the silica optical fiber processing temperatures. In addition, the encapsulation with different materials, as well as the use of air cavities, can extend the temperature application range of such sensors’ devices [75]. In this approach, there is the application of sapphire wafers for the FPI cavity development [76]. Thus, the use of sapphire optical fibers is generally used in sensors devices for high temperature assessment, due to the temperature resistance that make them suitable for temperature applications in a range higher than 1000 °C, since the melting point of such materials is around 2045 °C [71].

The temperature sensors applications using optical fiber-based approaches are generally related to FBG sensors, due to their inherent sensitivity to temperature variations. In general, the FBGs are inscribed using UV lasers with holographic/interferometric/phase mask techniques [77], which typically result in type I gratings that operate in temperatures below around 450 °C, since higher temperatures lead to the erasing of the grating [78]. To address this issue in high temperature operations, the direct inscription using fs lasers result in the possibility of using such sensors in temperatures close to the ones of the material processing [79]. In addition, the annealing treatments in silica optical fibers for grating regeneration lead to changes in the FBG that enable its applications in temperatures higher than 1000 °C, due to the changes in the optical fiber material and grating structures [78]. Another straightforward approach for FBG-based high temperature sensing is the use of sapphire optical fibers, due to their high temperature resistance. To that extent, fs lasers were used in the FBG inscription in sapphire fibers using direct inscription [80] and phase mask [81] inscription methods, which result in a temperature sensor able to withstand temperatures higher than 1500 °C.

### 3.2. Pressure Sensing

Pressure assessment is critical in different fields for structural condition monitoring, environmental assessment, control units, and health monitoring. Therefore, pressure assessment is used in applications ranging from industrial measurements [82] to medicine [83] and biomechanics [84], just to name a few. In aerospace, pressure sensing can influence the predictive maintenance and optimization of its costs [85], structural health monitoring of aerospace and aeronautics assets [86], fuel economy, and even in the flight navigation [87].

The pressure measurements in engines stages of aircrafts are an important field of investigation, where the pressure assessment in turbine airfoils, as well as the effects of position and dynamics variations in aerodynamic structures, can be obtained [64]. In addition, the pressure assessment in the cabin and cockpit is related to the safety of the crew members and early detection of components malfunction [23]. Figure 4B presents the schematic representation of the regions and components of aircrafts for pressure sensors applications, where it is also important to mention that some of these regions are subjected to high temperatures (up to 800 °C) and such issues also increase the demands of pressure sensors for high temperature operation [87].

Considering the applications and physical properties related to the pressure sensing, turbulence is an important phenomenon with some unsolved features for quantitative predictions of structures under turbulent flow [87]. In this case, there are variations in the pressures and velocities, which need to be dynamically and precisely measured, since they result in the possibility of obtaining temporal and spatial variations related to the Reynolds number in turbulence [23]. Despite the large dimensions of aircrafts, the aerodynamics of their components generally result in a microfluidics study, since there is the necessity of thin boundary layers investigation to evaluate critical effects, such as flow separation and friction drag [87]. The pressure assessment in such small layers is important on the assessment of such effects for proper design and mitigation.

The optical fiber-based sensors for pressure sensing generally employ the advantages of small dimensions, flexibility, and multiplexing capabilities of such devices for their embedment in different structures, considering a variety of geometries and configurations [62]. In this context, the assessment of mechanical parameters (e.g., pressure, force, and displacement) usually requires the integration of the optical fiber sensor in different structures, which include cantilevers [88], diaphragms [89], or platforms [90]. In general, diaphragm-embedded structure lead to a compact sensor device, with the possibility of customizing the sensor performance using different diaphragm materials, geometry, and assembly methods [91]. In the diaphragm configuration, there are two major geometric assemblies for the optical fiber integration in such scenarios, where the diaphragm can be positioned on the tip of the optical fiber [92] (perpendicular configuration) or along the optical fiber (parallel configuration) [93].

Considering the case with the diaphragm on the tip of the optical fiber, the advantages are the possibility of a miniature sensor development and the possibility of high-resolution measurements, where the diaphragm has the same dimensions as the cross-sectional area of the optical fiber [94]. In this configuration, the intensity variation-based sensors and interferometers (especially the FPIs) are used with micromachined flexible diaphragms positioned in a hollow structure [91]. In the case of FPI sensors using the diaphragm on the tip of the optical fiber, there is an extrinsic FPI formed in the region between the optical fiber tip (which is used as a reflector) and the tip of the diaphragm that can also include a reflective surface [95]. In this case, the sensor sensitivity is related to the diaphragm mechanical properties, since the spectral variations on the FPI are due to the cavity length variation with the diaphragm deformation. Thus, the use of flexible diaphragms with elastomers or other materials lead to a highly sensitive device, as discussed in [96], which make them suitable for the measurement of small pressure variations. In addition, the possibility of increasing the dynamic range, as well as the possibility of measuring higher pressures, is achieved by optimizing the diaphragm material properties, which make it suitable for gas pressure sensing [92]. Despite the difficulties and higher demands on the fabrication tolerance of such small diaphragms, the configuration using diaphragm on the tip of the optical fiber also inhibit the multiplexing capability of the OFS using a single fiber cable, since there is only one diaphragm at each fiber [91].

In another configuration for optical fiber pressure sensors, the diaphragm positioned along the optical fiber enables the development of diaphragm-embedded OFS based on intensity variation [97], interferometers [98], and FBGs [99]. Due to larger dimensions of the diaphragm in this configuration, the fabrication tolerances are smaller (when compared with the diaphragm on the tip of optical fibers) and the multiplexing capabilities are favorable, since many diaphragms can be employed along the optical fiber [100]. It is also important to mention that such a configuration leads to the possibility of positioning the optical fiber in different regions of the diaphragm, considering different planes, i.e., related to the position on different thickness [101], as well as the transverse area positioning, such as the fiber in the edge of the diaphragm [102]. For these reasons, it is possible to use this approach on the development of distributed systems for density profiling [103] and pressure mapping [104]. The operation principle of such sensors is based on the diaphragm strain, due to the pressure applied on the sensor assembly, which is transmitted to the optical fiber, leading to spectral variations in the sensors [82]. To that extent, not only the diaphragm properties are important on the sensors’ performance, but also the optical fiber mechanical properties, since the sensor is based on the strain transmitted to the optical fiber. Thus, the use of POFs generally leads to higher sensitivity and resolution in the pressure sensors, due to their lower Young’s modulus (when compared with conventional silica optical fibers) [99].

It is worth mentioning that such pressure sensors, irrespective of the configuration, are generally sensitive to temperature variations, not only due to inherent temperature sensitivity of the OFS, but also the thermal expansion and mechanical properties variations of the diaphragms [105]. In addition, there is a temperature variation on aircraft applications, as mentioned above, which increases the demands of temperature insensitivity on the OFS for pressure sensing applications. For this reason, different temperature compensation techniques have been proposed throughout the literature [106]. Such techniques include the use of a temperature sensor without pressure sensitivity to obtain a temperature reference system, which is compared with the results of the pressure sensor using the direct difference between both sensors signals considering their sensitivities. This approach can be used with different OFS approaches including FBGs (uniform and non-uniform) [107], different interferometers [108], and intensity variation-based sensors [109]. Furthermore, the use of mechanical structures for the development of temperature-insensitive pressure sensors, which include the application of a metallic sheet in the diaphragm region for the positioning of the temperature compensation/reference system [110]. It is also possible to position two FBGs in the same diaphragm for the simultaneous assessment of pressure and temperature, where the sensor system is characterized as a function of the pressure and temperature prior to its application in a real scenario, in which the temperature and pressure are simultaneously varied [111].

The intrinsic or extrinsic interferometric cavities along an optical channel generate an interferometric sensor [51,112]. Interferometric sensors with practical applications include FPI sensors and low coherent interferometric sensors (called as SOFO interferometric sensors) [113]. An FPI sensor may have a resolution as high as 0.15 με, a strain measurement range of ±1000 με that may be expanded to ±5000 με, and the capacity to function at temperatures ranging from 40 °C to + 250 °C. FPI sensors are extremely compact, ranging in length from 1 mm to 20 mm, and can be incorporated in certain structural components without incurring any weight penalty or negative impacts. However, its low multiplexing capacity is a disadvantage.

It has been stated that SOFO interferometric sensors are the most successful low coherent interferometric sensors for structural health monitoring (SHM), having been successfully placed in hundreds of structures, including bridges, buildings, oil pipes, and tunnels. SOFO interferometric sensors are long-gauge sensors, in contrast to FPI sensors. They have a measurement range beginning at 0.25 m and extending to 10 m, or even 100 m, with a micrometer-level resolution and temperature insensitivity, high precision, and stability. However, they can only measure elongations and contractions at low speeds (0.1 Hz–1 Hz) and are unable to detect impact damage in aircraft structures.

There are three types of distributed fiber optic sensors: Rayleigh-based optical time-domain reflectometry (OTDR), Raman-based optical time-domain reflectometry (ROTDR), and Brillouin-based optical time-domain reflectometry (BOTDR).

OTDR is the first generation of distributed fiber optic sensors employing Rayleigh scattering to reflect the attenuation profiles of long-distance optical fiber networks [114]. An optical pulse is introduced into an optical fiber link, and the power of the Rayleigh backscattered light is measured by a photodetector as the light pulse propagates along the fiber link. This measurement is typically used to determine fiber loss and break locations, as well as to evaluate splices and connectors.

In recent years, ROTDR and BOTDR have been utilized for distributed sensing applications. Their operation methods rely on the nonlinearities of optical fibers, which generate additional spectral components. These additional spectral components are impacted by environmental conditions external to the system. Consequently, changes in external measurands can be determined by evaluating the spectral content appropriately. ROTDR is based on the Raman scattering phenomenon, which generates both anti-Stokes and Stokes components [115]. As the fiber connection itself is the sensor, the intensity ratio between these two components can provide temperature information at any point along the fiber link. Since the amplitude of the Stokes components is independent of temperature, ROTDR can only measure temperature with a temperature resolution of 0.2 °C, and not strain. With a spatial resolution of 1 m, the sensing distance of ROTDR is typically restricted to around 8 km.

In BOTDR, light is partially scattered back based on Brillouin-scattering phenomenon [116]. BOTDR can monitor both temperature and strain, since the frequency of the scattered light is dependent on the temperature and strain applied to the fiber link. The basic BOTDR measurement distance is 30 km and can be expanded to 200 km. The resolution ranges from 1 to 4 m.

For the purpose of increasing the service life of aging airplanes, the SHM of damaged aircraft panels fixed with bonded patches has garnered considerable interest. Using FBG sensors, the positions and forms of fatigue cracks and disbond fronts are identified in aircraft panels fixed with double-sided bonded patches [117,118]. The specifications, sensor performance, and other technical information are shown in Table 1.

## 4. Sensors for Navigation

**Figure 5:**
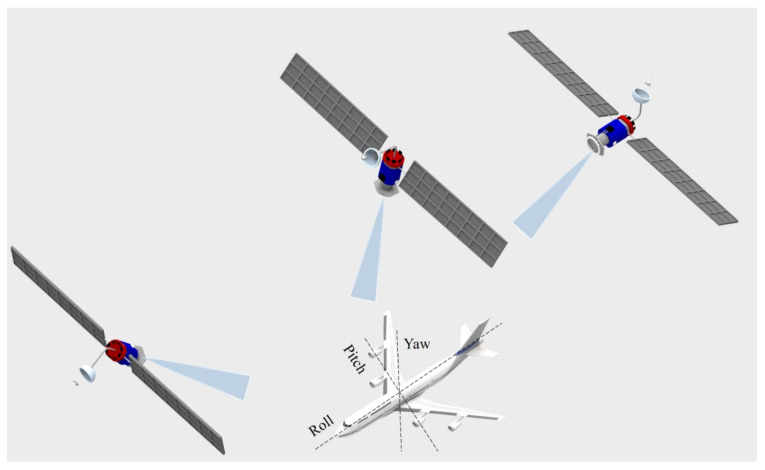
Inertial navigation system and global positioning of an aircraft.

Inertial navigation systems (INSs) are important systems for aircraft navigation that can also apply to personal navigation, car navigation, and unmanned aerial vehicles [119]. The continuous advances in autonomous vehicles and navigation systems place demands in the compactness, as well as the precision of such navigation systems [120]. These demands lead to developments in fiber optic gyroscopes (FOGs) and general MEMS in the development of systems with small errors in the position and attitude, due to the reduction of the sensors uncertainties and nonlinear signal processing approaches [121]. In this context, calibration methods and automatic error corrections increase the accuracy and general performance of INS. As another common approach for the development of reliable navigation systems, the INS can be integrated with the Global Positioning System (GPS), where the GPS enables the calibration and reduction of bias in INS [119]. In addition, the fusion between the INS and GPS enables the tracking performance of the GPS [122]. Such improvement is achieved using error calibration techniques based on feedforward or feedback methods. Moreover, the integration can be achieved by means of using only the GPS for the position and velocity calculations, whereas the navigation filters estimate the position, velocity, and attitude from the INS and the GPS’s position, and velocity data are used as the reference for calibration of the INS [119]. Figure 5 presents a general schematic about inertial navigation and global positioning systems on an aircraft.

One of the sensors for the navigation system is the displacement sensors, where conventional linear variable differential transformers (LVDT) and rotary variable differential transformers (RVDT) can be employed [23]. Such displacement sensors are capable of operating in high temperature ranges (from below 0 °C to higher than 200 °C) and of the possibility of positioning in different regions of the aircraft, but at the cost of complex signal processing. To that extent, the development of optical fiber-based displacement sensors can address some of the issues of the electronic sensors, such as magnetic field immunity and potential applications in higher temperatures conditions [123].

Among the sensors for the inertial navigation systems, the continuous improvements in gyroscope technologies and rotation measurements are critical for the evolution in navigation systems [124]. In this case, the rotation rate indicates the variations in the heading and attitude of the system. Moreover, the accelerometers are also important in the navigation system for the assessment of acceleration amplitude and direction. If the gyroscopes are considered, such devices are positioned on a frame (or mechanical structure) to obtain the angular velocity of the rotating structure [125]. A typical classification of the gyroscopes includes the mechanical gyroscopes, optical gyroscopes, and MEMS gyroscopes [125]. However, we focus on the optical gyroscopes in this review. The development of the optical gyroscopes is motivated by sequential breakthroughs in optoelectronic technologies [126], which include the widespread of optical fiber technologies. A FOG is an angular velocity sensor using optical fibers, where the Sagnac effect is used for the assessment of the rotational rate by considering the advantages of high sensitivity, temperature, and pressure resistance, in conjunction with the well-known electromagnetic field insensitivity [127]. For these reasons, FOGs are widely used in general navigation systems, especially in aviation [128] and aerospace [129] applications.

Another approach for FOGs is based on the resonant structures, resulting in the so-called RFOGs [124]. In the last years, the use of novel optical fiber structures, e.g., the use of photonic crystal fibers (PCFs), has accelerated in order to obtain structural birefringence in the sensing structure [130]. If a hollow core PCF is used, the RFOG can even result in smaller cross-sensitivity as a function of temperature in conjunction with the lower backscatter and nonlinear effect, which increase the polarization noise controllability of the sensing structure [131]. For these reasons, the use of optical components in the coupling of hollow core PCFs with the resonator structure was proposed in [131], resulting in a high temperature stability of the device. In addition, a high finesse can be obtained in the resonant cavity using a PCF coupled with a single mode fiber, resulting in a hybrid PCF resonator structure, where such a structure resulted in a gyro bias stability of 0.5°/s [132]. It is also worth noting that the use of hollow-core PCF in the gyroscope prototype can reduce the polarization crosstalk of the structure [133]. Furthermore, the use of hollow-core anti-resonant fiber (NANF) structure resulted in the extended performance of the RFOGs, with the possibility of using a frequency differential RFOG structure to obtain a stability as high as 0.05°/h [134].

Optical fiber-based accelerometers have an even higher developments and applications, since they are not only used in navigation systems, but also in structural health monitoring [135] and physiological parameters monitoring [136]. To that extent, the optical fiber-based accelerometers were developed using different OFS approaches, as thoroughly discussed in [137]. The simplest approach for optical fiber accelerometers is the use of the intensity variation principle, where the transmitted optical power variation is analyzed as a function of the acceleration [138]. In this case, the sensor can operate in the coupling principle, where a fiber is positioned in a light source, whereas another fiber is positioned in a photodetector [139]. If this configuration is used, one of the fibers is isolated from the vibration/acceleration variation, and the other end is connected to the system with proof mass for the vibration transmission [140]. Some important drawbacks of this approach are that the sensors are sensitive to environmental variations and present low precision, due to the high sensitivity to misalignments.

As higher precision for optical fiber-based accelerometers, the interferometric-based accelerometers are developed using cavities, generally based on FPIs [137]. In general, cavity-based accelerometers use a movable structure connected to the cavity, in which the cavity length varies as a function of the acceleration. In this case, a sub-nanometer potential resolution can be achieved using such a configuration [141]. The application of micro-optical-electro-mechanical systems (MOEMS) resulted in the novel configurations for the optical accelerometers that can further increase the accelerometer performance [142]. In this approach, there is a microscale proof mass and a silicon frame to create the accelerometer structure, where the displacement and acceleration are obtained from the spectral features’ variation of the cavity, with the possibility of tuning the accelerometer parameters (such as proof mass and stiffness) to achieve high resonant frequencies and small noise. In another MOEMS accelerometer, the bioinspired shape was proposed in [143], where a battery was used as proof mass and the sensors were embedded in a transparent web-like structure for movement analysis and highly sensitive displacement measurements.

One of the most common optical fiber-based approaches for accelerometers development is the integration of FBGs in mechanical structures [144]. In this context, many different approaches using single [145] and double [146] cantilevers, as well as diaphragm [147] and flexible hinge [148] structures, were proposed. This approach is based on the strain produced in the FBG, due to the inertial displacement of the proof mass under acceleration [149]. For this reason, it is possible to develop 2- and 3-axis [150] accelerometers using different assembly conditions and using the multiplexing capabilities of the FBGs. Thus, it is possible to develop FBG-based accelerometers for multipoint measurement, which play an important role not only in navigation systems, but also in structural monitoring in aircrafts. Furthermore, such devices are able of measuring tilt angles by means of embedment in mechanical structures and using different types of FBGs, such as the tilted FBGs [151] that can measure such parameters, with the additional advantages of self-referencing signals using the spectral features of the gratings. Such gratings-based devices can also include additional information for the navigation systems and enable novel data fusion approaches for ever higher accuracy and reliability of in-flight data.

## 5. Pilot Vital Health Monitoring

There are over one million active pilots in the world, with over two-thirds residing in the United States. The average age of pilots is increasing across all aviation industries. Due to aging, pilots over the age of 50 may be more prone to accidents than pilots in their 30s. In addition to mechanical and system failures, certain examinations have shown that pilot error is a major cause of accidents. As a result, the Flight Safety Foundation identified cognitive health as a crucial component in pilot safety. Subsequently, risk classification and user experience outcomes validate an immersive virtual reality (VR) cognitive screening and intervention tool for elderly pilots [152]. This VR simulation tool serves as the basis for this cognitive evaluation, since it provides a portable, cost-effective, and dependable approach for evaluating pilot cognition [153].

Today, the aircraft engine and flight control systems are equipped with a number of sensing units for determining the pilots’ normal health condition [154,155,156,157]. Recent variations of the infectious severe acute respiratory syndrome coronavirus 2 (SARS-CoV-2) cause coronavirus disease 2019 (COVID-19) pneumonia, which has afflicted the entire world. It mostly affects the aviation industry and poses a severe risk to pilots. Numerous problems are observed in pilots infected with COVID-19, including neurologic issues during the acute phase, following recovery, and even after immunization. COVID-19 patients have been reported with a variety of neurologic complications, including encephalitis, encephalopathy, stroke, headache, loss of smell and taste, dizziness, seizures, refractory status epilepticus, myelitis, myopathy, acute disseminated encephalomyelitis, leukoencephalopathy, Kawasaki syndrome, Guillain–Barré syndrome, and neuroleptic malignant syndrome [158].

In a recent case study, a 43-year-old helicopter pilot was sent to the emergency department, due to influenza-like symptoms. After receiving treatment for COVID-19, he recovered and returned to flying. During flight, pilots experienced moments of dizziness and unconsciousness. Although the co-pilot salvaged the helicopter, it has been determined that robust sensory elements are required to identify the pilots’ present health condition during flight operations [158].

Monitoring the pilot’s vital signs is essential for ensuring the safety of an aircraft and its occupants. In this instance, a cockpit equipped with a health monitoring system provides a means for both the occupant and the ground station to be aware of the pilot’s normal health state throughout the flight. Multiple types of sensors, such as a pulse oximetry sensor, are positioned at a preset area of the pilot seat that is within reach of the occupant. The sensor for a pulse oximeter is put at a preset, stable point for the insertion of a finger, which offers a satisfactory response to the sensor while the individual is seated. The sensor is linked to the communication link via a control box that aids in data processing and presents the data in an appropriate format on the cockpit’s multifunctional display system. Throughout the flight, the pilot can enter his or her finger into the sensing unit to monitor his or her pulse oximetry and view the parameters on the multifunctional device. Similarly, the Republic of Singapore Air Force (RSAF) adopted the search-and-rescue (SAR) and heli-medevac-based services in 1971 for the Singapore flight information region, in which the flight doctor and a medic are supplied for medical care on board the helicopter [159].

Researchers from the United States Air Force are investigating methods for determining the health of pilots in high-performance aircraft. They intend to develop a technology that monitors and notifies pilots to potential problems with physiological states that affect pilot performance during flight, such as a sudden drop in oxygen concentration. In this work, emphasis must be placed primarily on integrating and experimenting with existing sensor technologies, as well as doing the evaluation. Researchers are interested in integrating sensors and monitoring many parameters, such as flight environment monitoring, sensor fusion, monitoring of pilot vital signs and respiratory function, data storage and processing, onboard analytics, and pilot alerts. To develop integrated sensors, multiple hardware components are necessary. The employed hardware is small, lightweight, and self-powered enough to be easily integrated into the cockpit of an aircraft. This type of hardware system consists of smart phones/tablet computers and embedded computing that facilitate wireless networking, such as Bluetooth low energy. The in-line sensor technologies were developed for measuring the air-quality and supply the aircraft life-support systems. The air quality consists of the appropriate level of carbon dioxide, oxygen, flow, pressure, and similar impurities. Similarly, pilot vital health monitoring system incorporates the sensor systems to provide direct or indirect evidence of pilot blood or tissue oxygenation, blood circulation, peripheral capillary oxygen saturation (spO2), respiration rate, and estimated core temperature. U.S. Air Force scientists also developed a unique cockpit sensor for the measurement of their any difficulty in the abilities function and alert to the pilots to behave properly during flight in high-performance aircraft. Researchers are trying to develop and showcase existing sensor technologies to help keep pilots functioning and feeling good in the cockpit. They are looking for self-contained and self-powered sensor hardware that is small enough to fit into airplane cockpits.

**Figure 6:**
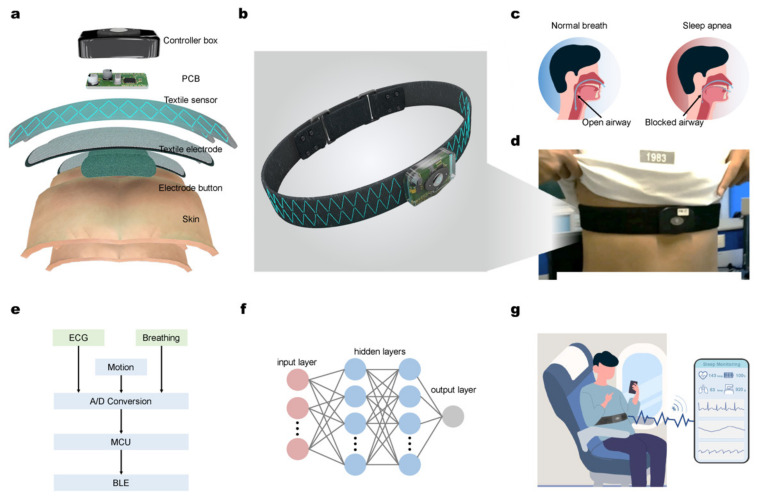
Seat belt point-of-care system in an airplane, (**a**) illustration of our developed chest band’s layered hardware structure, and (**b**) the produced chest band device, (**c**) difference in aspiration between normal breathing and sleep apnea produced by airflow, (**d**) photographs of a human person wearing a chest belt, (**e**) a flowchart of the monitoring system’s operation, including hardware and software, (**f**) a neural network used as a classifier in the system. In the network, the red nodes represent the input layer, the blue nodes represent the hidden layers, and the gray nodes represent the output layer, (**g**) diagram of an airline point-of-care monitoring system in the user’s client, with Bluetooth data transmission to the mobile terminal for hybrid physiological signal detection (adapted from [160]).

The International Civil Aviation Organization (ICAO) defines fatigue as a physiological state of reduced mental or physical performance capability caused by sleep deprivation, protracted awake, circadian phase, and/or workload (mental and physical activities). This impairs the pilot’s attentiveness and ability to perform safety-related operations correctly [156]. A proper pilot monitoring system in an aircraft consists of the plurality of sensors arranged to monitor several health parameters of the pilot, for instance, the aircraft state monitoring system is used to monitor the flight situation data of the aircraft, and the analysis system is used to determine the incapacitation level of the pilot for health parameters. Thereafter, human machine interface utilized to interface with one or more processors to offer an interface between the pilot and pilot monitoring system. Then, the human machine interface helps to notifies the pilot based on the determined incapacitation level. As illustrated in Figure 6a–g, the system’s hardware design includes a controller box, printed circuit board (PCB), textile electrode, and electrode button on the surface of human skin [153].

## 6. Novel Nanomaterials for Aerospace Applications

Due to their unique properties, such as high-aspect ratio, large surface area, tailorable electrical and thermal conductivities, high anisotropy, and distinctive optical properties, the synthesis and characterization of nanomaterials (100 nm in size) have been the primary focus of academic, private, and government organizations for the past two decades [161]. These characteristics lead to the development of lightweight, high-strength, multipurpose structures, efficient energy storage systems, strong radiation shielding, and cutting-edge optical fiber sensors [162].

Trying to align the reason for biomolecular research based on nonmaterial-assisted optical fiber sensors helps the reader in understanding the need for sensor development for aeronautical applications. One of the most severe human diseases, diabetes, can be caused by high blood glucose levels [163]. Diabetes is characterized by elevated blood sugar levels that result in consequences such as renal failure and diabetic retinopathy [164,165]. In diabetes diagnosis, the food industry, biotechnology, and aerospace, glucose sensors play a significant role. In these works, gold nanoparticles, graphene oxide, and MXene nanomaterials were primarily used to develop optical fiber sensors for glucose biomolecule detection.

Acute myocardial infarction (AMI) has emerged as a leading cause of death on a global scale. In this work, cerium oxide nanoparticles were used to develop an optical fiber sensor for cTnI detection [166]. Within 1.5 to 3 h of symptom onset, acute myocardial infarction can cause irreversible myocardial damage and blood troponin release. An important biomarker of AMI is cardiac troponin I (cTnI). Creatinine is a byproduct of human muscle metabolism and an important clinical indicator of diabetes, kidney disease, kidney failure, and muscle wasting [167,168]. To lower the incidence of renal disease, the early detection and prevention of elevated creatinine levels are crucial. These sensor probes feature graphene oxide, gold nanoparticles, molybdenum disulfide nanoparticles, and niobium carbide MXene nanomaterials. Cholesterol is an essential part of the human body. It is created by the liver and is also a part of a healthy diet because it is a precursor to extremely vital biological substance. Abnormal levels of cholesterol in the blood can lead to excessive blood pressure, heart disease, coronary artery disease, cerebral thrombosis, atherosclerosis, and a number of other common conditions [169,170]. These optical fiber sensor probes are developed utilizing gold nanoparticles, silver nanoparticles, and zinc-oxide nanoparticles. Alanine aminotransferase (ALT) is a serum aminotransferase whose activity must be determined to diagnose and assess liver disease. In this study [171], molybdenum disulfide nanoparticles and cerium oxide nanoparticles are combined to increase the performance of an OFS. When certain metabolic conditions, such as obesity, high-fat diabetes, and other symptoms, are present in the body, clinical trials and laboratory tests have demonstrated that ALT levels may be somewhat raised. In addition, alanine transaminase activity leads to somewhat increased alanine transaminase levels in a range of muscle disorders, including viral hepatitis and muscular dystrophy. Uric acid (UA) is an extremely significant component in human serum and urine [172]. The concentration of UA in the blood can be increased by a lack of exercise, a poor diet, and improper drugs [173]. Excess uric acid can produce solid urate in the body, which can lead to catastrophic conditions, such as gout and kidney stones [174].

Similarly, wearable sensors are ground-breaking health monitoring gadgets that enable continuous monitoring of physical and biological characteristics. Recent paper [175] have detailed the development of innovative optical sensors for wearable vital health monitoring devices. A detailed discussion of the substrates, sensor platforms, and biofluids was utilized for the detection of target molecules.

In this way, low-cost wearable technologies could improve the quality of health monitoring systems and permit continuous and early disease diagnosis in aerospace applications.

In addition, and not less importantly, it is obvious that advanced materials, such as thermal protection systems based on carbon–carbon composites and turbine blades based on metal alloys, play a significant role in aircraft applications. Multiple industries have begun to develop improved airplane materials. Single-crystal nickel-based turbine blades and aluminum bulk alloys are examples of applications for nanomaterials. Research and development evolved and broadened its applications to encompass, among others, radiation protection, thermal protection, structural nanostructures, space propulsion, electronics, and sensors. In numerous applications, carbon nanostructures (nanotubes, CNTs, and graphene) and inorganic nanomaterials are used (silica and metal oxides). Subsequently, customized nanoparticles enabled significant improvements to the structural and non-structural components of virtually all space and aviation systems. It primarily provides a reduction in weight, maintenance of mechanical strength, increased adaptability, multicomponent monitoring, storage, and transmission, efficient power production, greater radiation protection, and long-term life support for exploration. The advances in the synthesis and characterization of carbon nanostructures (CNSs) and carbon nanotube nanosheets (CNT nanosheets) produced on carbon fibers provide new opportunities for using the multifunctional features of these materials in aviation components. Several scientific studies suggested that CNT-based coatings, films, and sheets facilitate electrostatic dissipation and electromagnetic interference shielding with low effort [176].

There are vacuums, severe temperatures, space debris, micrometeoroids, and significant variances owing to the sunspot activity in the space environment. These criteria determine the design and construction of aircraft systems and spacecrafts primarily. The system’s performance degrades when exposed to atomic oxygen. The special materials used in aircraft applications can withstand high temperatures and reduce the erosion yield factor [177].

Due to high-phase temperatures, aeronautical structures require materials with high strength and stiffness for mechanical aspects [178]. This can be accomplished by the use of composites containing a blend of graphene and epoxy resin as a curing agent to produce the desired results in aeronautical applications. It boosts the base material’s strength and temperature resistance, hence enhancing the material’s tribological behavior. Thus, improved composites contribute to the development of lightweight aircraft constructions with corrosion and fatigue resistance [179].

Carbon and glass fiber composites are also utilized in the production of sensors mounted on aircraft SHM [180]. Prior to that, in 1940, Bower was the first person to employ the nanocomposite technology [180]. Typically, nanocomposite consists of nanoparticles embedded in a reinforcing metal matrix material composed of Al/SiC and Al/BN [181]. This technology introduces the qualities of robustness, portability, and affordability. Compared to copolymers, the nanocomposites have a greater pyroelectric coefficient. Due to their low density and strong damage resistance, these materials also offer a lengthy service life. The behavior of tribology in aeronautical constructions is determined by severe temperatures. In these nanocomposites, the function of alumina particles is to minimize the resistance to wear and friction.

**Figure 7:**
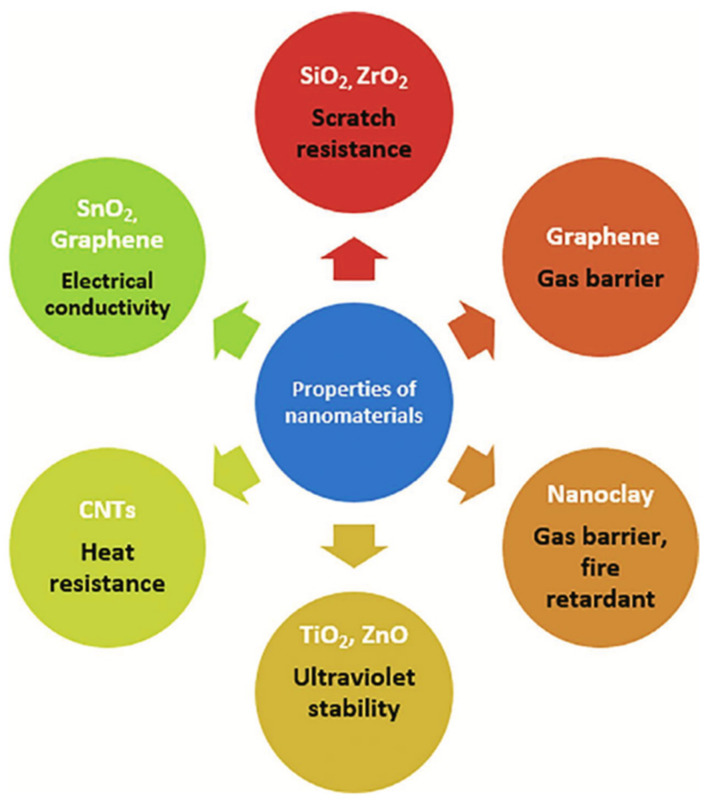
Properties of nanomaterials for aerospace applications (adapted from [183]).

Nanocomposite materials, such as alumina and polytetrafluoroethylene, can be utilized to optimize the performance of existing advanced structures. Carbon nanotubes (CNT), carbon nano-beads, multi-walled carbon nano-tubes (MWCNT), carbon nanorods, diamond-like carbon, carbon nanofibers, and carbon nanocones are present in the nanocomposite materials [182]. The performance of these materials exceeds that of conventional materials. MWCNTs, CNTs, graphene oxide, and polymer-clay nanocomposites are the most important nanocomposites for aerospace industries [183,184], as shown in Figure 7.

Similarly, molybdenum disilicide nanoparticles combined with an aluminum matrix play a significant role in aerospace and provide wear resistance to prevent degradation [185]. Through the use of nanocomposites based on high-strength titanium-based materials, it is possible to achieve superior aerospace qualities. The nanocomposites of graphene oxide and titanium nanopowders can be employed as the matrix system reinforcement, in order to offer a high hardness, which is often used in a number of aerospace structural components [186].

**Figure 8:**
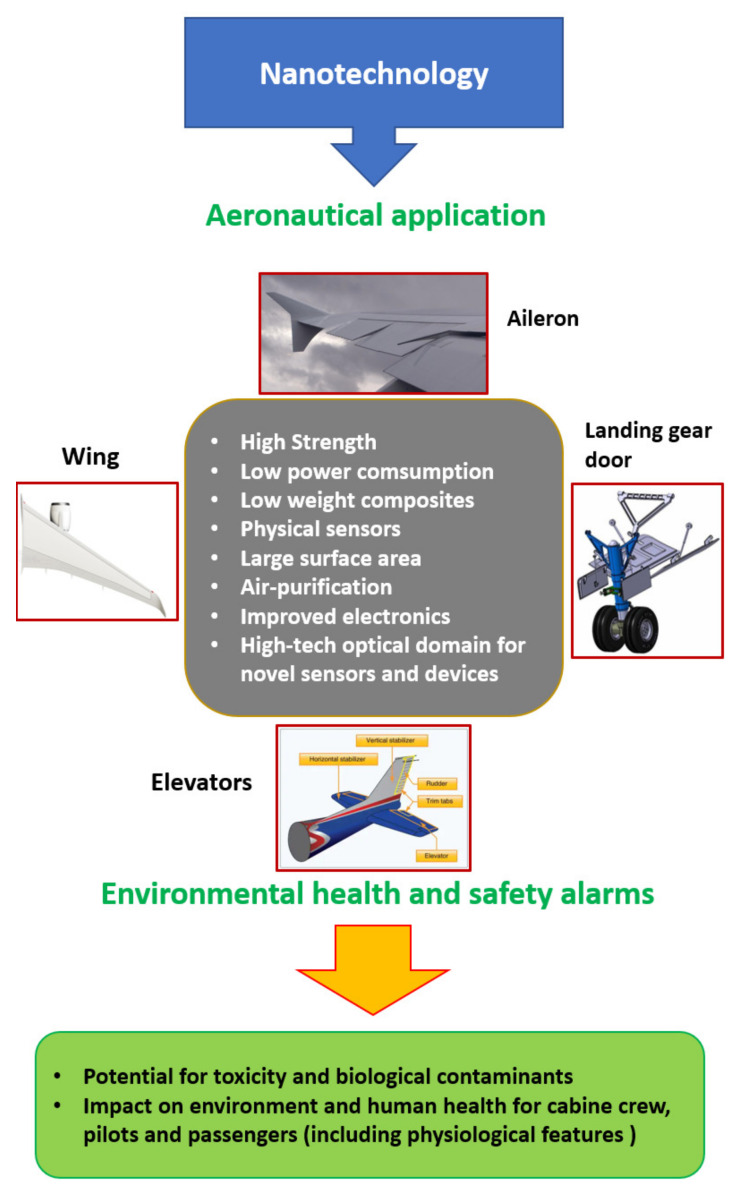
Applications of nanomaterials in the aircraft industry.

Using laser sintering, the GO may be simply and rapidly dispersed throughout the matrix. Due to the development of multifunctional materials, space exploration has accelerated in recent years. Using polymer nanocomposites with CNT sheet reinforcement, the vibration damping factors can be decreased. MWCNTs have distinctive electrical, mechanical, and thermal qualities that are advantageous for aeronautical applications [187], as shown in Figure 8.

These nanocomposites also enable the aerospace industry to withstand sub-zero and high temperatures, enabling them to resist the severe conditions of lower earth orbit and outer space [188]. CNT is widely used in aerospace applications as a result of its significant properties. Similarly, the advancement of Al-, Cr/Al-, and Cu/Al-coated advanced structures boosts heat and adhesive resistance [189].

Nanocomposites provide the optimal technology for aircraft applications, due to the intertwined nature of sustainable impacts and constant generational advancement. By incorporating nanocomposites into complex aeronautic designs, waste creation during the manufacturing process can be reduced. This also facilitates the creation of low-maintenance, lightweight structures [190].

## 7. Future Perspectives

As is well-known, the enlarged use of composite structures in the aeronautical industry started over the past few decades with newer aircrafts made largely of composite material and produced many new fruitful contributions to the progress of the SHM area for composite aeronautical and aerospace structures. Applying SHM in the aeronautical and aerospace industries have not yet fully matured, and their applications to composite structures are particularly challenging. In terms of a realistic aircraft maintenance scenario, such requires a full damage diagnostic on all four SHM levels, which means damage detection, damage localization, damage type identification, and damage severity. Presently, the scientific, industrial, and end-user communities generally view optical sensors as a technology with the highest potential for the continuous real-time monitoring of aircraft structures and, not less importantly, the tracking of some critical biochemical parameters, as well as destructive contaminants.

The optical sensors, which can include optical fiber-based sensors, have also found applications in feeding back real-time measurements of weight distribution, reliable aviation fuel gauging sensors, or even water content detection and online monitoring in aviation fuel. Further, the distributed optical sensors are able to test the structural integrity of the wings and fuselage (following the four SHM levels mentioned), as well as judge the performance of the engines, icing on the wings, loading on the landing gear, and very importantly, the cockpits environment (not only in commercial aircrafts, but also military ones) [191,192,193]. In this way, with the continuous development of autopilot systems and flight assistance systems, pilot performance and air safety from the cockpits (temperature, humidity, pressure, and also detecting biological contaminants aboard aircrafts, for example) need to be improved significantly. Pilot behavior recognition, based on multi-modality fusion technology [194], using physiological features acquired online, as well as critical parameters like fatigue, hypo/hyperoxia, surges of acute stress level, and/or a sudden loss in blood flow [161,162,163,164,165,166,167,168,169,170,171,172,173,174,175,195,196,197] of pilots, is crucial to obtain in real-time data and process them via an integrated smart tool. 

In additional, nanofilms, nanofibers, and nanoparticles demonstrate a variety of advanced capabilities, including good thermal and electrical properties, safer coating, cleaning, corrosion resistance, and potential toxicity facilities in a variety of disciplines of aviation components. The surface coating can also safeguard aircraft components from severe hazards. In addition, it improves the unique advantages and performance characteristics, compared to the standard metals and composites used in the production of many types of aircraft components. The incorporation of nanomaterial structures and nanomaterial-based devices into the manufacturing of aircraft helps their maintenance and repair, consequently reducing the operational costs, and it is quite important to consider them not only for aircraft components improvements, but also to progress in terms of the physiological features of the cabin crew, pilots, and passengers.

## 8. Conclusions

The building blocks of future aircrafts (both commercial and military) are in place, allowing for the next phase of aerospace development to attain significantly higher levels of sustainability in the aviation industry, while reducing its carbon footprint, with the help of lightweight technologies such as OFS. Increasing public concern about climate change, coupled with government support of green projects, should foster innovation in this field, and optical technology can be a viable alternative for the continued expansion of such a vital industry. The employment of OFS and innovative nanophotonic materials in aircrafts to enhance the safety of the aviation sector, as well as the safety of pilots and passengers, is strongly encouraged. As described in the article, the development of this industry will continue concurrently with the development of technologies fed by the optical domain, due to their distinct features.

## Figures and Tables

**Table 1:** Performance evaluation of selected fiber optic sensor technologies for aircraft monitoring.

	FPI Sensors [112]	SOFO Interferometric Sensors [113]	OTDR [114]	ROTDR [115]	BOTDR [116]	FBG Sensors [117]
Sensor type	Point	Long gauge	Distributed	Distributed	Distributed	- Point - Semi-distributed
Main sensing parameters	- Temperature - Strain - Rotation - Pressure	- Deformation - Strain - Force	- Fiber loss - Break location	- Temperature	- Temperature - Strain	- Temperature - Strain - Rotation - Pressure
Multiplexing	- Parallel - Time-division	- Parallel - Time-division	Distributed	Distributed	Distributed	- Quasi-distributed - Wavelength-division
Measurement point in one line	1	1	Depending on the range and resolution	Depending on the range and resolution	Depending on the range and resolution	10–50
Typical resolution strain (μStrain) temperature (°C)	0.15 0.1	1 N/A	N/A N/A	N/A 0.1	20 0.2	1 0.1
Capability for large wavelength shift detection (~10 nm)	Yes	No	No	No	No	Yes
Spatial resolution	0.1	0.1	1–10	1	1	0.1
Capability of fast response for acoustic signal detection (>100 kHz)	Yes	No	No	No	No	Yes
Advantages	- High sensitivity - Accurate	- Long gauge - High spatial resolution	Wide applications	- Infinite sensing points - Fiber integrated	- Infinite sensing points - Fiber integrated	- Linearity in response - Accurate - High resolution - Inherent WDM encoding
Disadvantages	Single point	Low speed (10 s)	Detection limitations	- Temperature only - High cost	Cross-sensitivity	Cross-sensitivity

## Data Availability

Not applicable.
